# Bio-electrical impedance vector analysis: testing Piccoli’s model against objective body composition data in children and adolescents

**DOI:** 10.1038/s41430-018-0292-x

**Published:** 2018-08-30

**Authors:** Jonathan C. K. Wells, Jane E. Williams, Rina Y. Quek, Mary S. Fewtrell

**Affiliations:** Childhood Nutrition Research Centre, Population, Policy and Practice Programme, UCL Great Ormond Street Institute of Child Health, 30 Guilford Street, London, WC1N 1EH UK

**Keywords:** Physiology, Biomarkers, Nutrition

## Abstract

**Background/Objectives:**

Bio-electrical impedance (BI) analysis is a simple body composition method ideal for children. However, its utility in sick or malnourished children is complicated by variability in hydration. BI vector analysis (BIVA) potentially resolves this, using a theoretical model that differentiates hydration from cell mass. We tested this model against reference methods in healthy children varying widely in age and nutritional status.

**Subjects/Methods:**

We compiled body composition data from 291 children and adolescents (50% male) aged 4–20 years of European ancestry. Measurements included anthropometry, BIVA outcomes (height-adjusted resistance (R/H) and reactance (Xc/H); phase angle (PA)), and fat-free mass (FFM), fat mass (FM) and FFM-hydration (H_FFM_) by the criterion 4-component model. All outcomes were converted to age- and sex-standardised standard deviation scores (SDS). Graphic analysis and regression analysis were used to evaluate the BIVA model.

**Results:**

R/H and Xc/H declined with age in curvilinear manner, whereas PA increased linearly with age. R/H-SDS and Xc-SDS were negatively correlated with FFM-SDS, H_FFM_-SDS. and FM-SDS. PA was positively correlated with FFM-SDS but unrelated to H_FFM_-SDS and FM-SDS.

**Conclusions:**

While previous studies of adults with major fluid perturbations support the BIVA model, it is less successful in predicting variability in FFM in healthy children and adolescents. BIVA outcomes varied as predicted by the model with H_FFM_, but not as predicted with FFM. Variability in adiposity also explains some of the variability in BIVA traits. Further work is needed to develop a theoretical BIVA model for application in paediatric patients without major fluid disturbances.

## Introduction

There is increasing interest in measuring body composition in children suffering from malnutrition or disease. Such measurements could potentially aid in diagnosis, guide clinical management, and help determine nutritional and fluid requirements [1]. Obtaining such data during early life could also help understand the long-term consequences of childhood disease. However, obtaining accurate body composition values is challenging in these groups, as the theoretical assumptions on which measurement techniques rely tend to be invalid [2]. Moreover, sick or malnourished children are unable to cope with demanding measurement protocols, and require simpler techniques.

Bio-electrical impedance analysis (BIA) is a simple bedside technique presenting few practical difficulties in sick or malnourished children, thus overcoming the second of these limitations. However, the conventional approach—predicting body water or lean mass from (height^2^/impedance)—is often inappropriate, due to the likelihood of altered body water distribution in many such children [3]. For example, some disease states are associated with dehydration, whereas malnutrition and other conditions such as renal disease or sepsis can also present with oedema.

An alternative BIA approach comprises vector analysis (BIVA) as developed by Piccoli [4]. Impedance (Z) is divided into its constituent components, resistance (R) and reactance (Xc), each adjusted for height (H). These components are plotted on ‘R/H-Xc/H’ graphs, in which data from a population are expected to form an ellipse, where one diagonal axis represents variability in hydration, and the orthogonal axis variability in body cell mass, a proxy for lean body mass [5]. Individual data points can be characterised by a vector, whose angle relative to the x-axis (calculated as [(Xc/R)×180°/π)] is termed ‘phase angle’ (PA) [4].

Piccoli’s approach is essentially qualitative rather than quantitative, with data in abstract ohms/cm units (R/H, Xc/H) or degrees (PA), but is proposed to indicate the level of both hydration and cell mass [5]. PA has been proposed to represent simultaneously a marker of cell mass and cellular health, hence providing a valuable index of clinical status, and numerous studies broadly support this hypothesis [6–9]. Higher values are proposed to reflect higher cell mass, cell membrane integrity and better cell function [10]. In one large study of adults, fat-free mass (FFM) was found to be the strongest predictor of PA [11]. Furthermore, major longitudinal changes in hydration correlate with changes in the ratio between R/H and X/cH, as for example in adults undergoing haemodialyses [12].

The position of the ellipse on the R/H-XcH graph varies in association with a population’s age, sex and range of body mass index (BMI), a broad marker of nutritional status [13], as well as ethnicity [14]. However there are some apparent inconsistencies between these findings and BIVA theory. As children grow they ‘mature’ chemically, one marker being a decline in the water content of FFM [15, 16]. On this basis, children would be expected to move upwards and to the right on the graph with increasing age. Contrary to this, however, studies show that R/H and Xc/H values decline with age [13, 17]. This results in a contrast between (a) data from populations demonstrating extreme variability in hydration [12, 18], which broadly support Piccoli’s model, and (b) data from normal healthy populations where variability in hydration arises through maturation [17], which appear not to support the model.

To date, however, no BIVA study in children has objectively measured the two parameters purportedly indexed by Piccoli's model (hydration and cell mass), and furthermore no analysis has attempted to control the raw data for age before applying the model. We collected BIVA and body composition data in a large sample of children and adolescents across a wide range of nutritional status. We generated age- and sex-adjusted standard deviation scores (SDS) for all variables, to remove these sources of variability, and then investigated the validity of Piccoli’s model in predicting variability in FFM and FFM hydration (H_FFM_), to test the conventional interpretation of BIVA data in healthy children.

## Methods

We conducted secondary analysis of data from two prior studies conducted by our group, both approved by the Ethical Committee of UCL Institute of Child Health and Great Ormond Street Hospital. Informed consent was obtained from all participants and/or their parents as appropriate. For this analysis, we included children of European ancestry only, as ethnicity has been associated with variability in fat and lean distribution [19–21], and the loci of BIVA ellipses [14].

Most individuals were from a study of healthy children/adolescents aged 4–20 years, recruited to establish body composition reference charts [22]. Inclusion criteria were the absence of any disease that might affect growth and development. No BMI exclusion criteria were applied, hence any child not recruited from an obesity weight-loss clinic was eligible. In addition, baseline data from obese children aged 7 to 14 years participating in weight loss intervention studies were used [23, 24].

Weight and height were measured in duplicate using electronic scales and a wall-mounted stadiometer. Body mass index (BMI) was calculated, and converted to SDS using UK reference data [25].

FFM, fat mass (FM) and H_FFM_ were measured using the 4-component model [26, 27]. Total body water (TBW) was measured using deuterium, assuming overestimation of TBW from proton exchange of 1.044 [28]. Total bone mineral content was measured by DXA, and body volume in duplicate by air-displacement plethysmography, with lung volumes predicted from child-specific equations [29]. H_FFM_ was calculated as TBW/FFM.

Single-frequency BIA was conducted at 50 kHz (Quadscan 4000 instrumentation; Bodystat, UK). This frequency is proposed to maximise signal-to-noise ratio and minimise frequency-dependent errors and variability of electric flow paths [30], though the optimal frequency also varies between individuals and by age [31]. Participants lay supine on a non-conducting couch. Disposable electrodes were attached in standard tetrapolar manner to left hand and foot. R, Xc and PA were recorded in duplicate, and the average used in analyses. R and Xc were standardised for height (H) and expressed as R/H and Xc/H in ohm (Ω)/m^4^. Prior to analysis, we excluded individuals with PA>8.0 (values in healthy people range between 5° and 7°, hence allowing for measurement error, values above 8° were considered implausible; *n* = 14 excluded) [10], as well as those with poor repeatability (exclusion criteria were duplicates >0.5 for PA, and ≥6.0 for R/H and Xc/H; *n* = 25 excluded).

### Statistics

FFM-SDS was divided into five categories: < −1.0 (*n* = 41); −1.0 to 0 (*n* = 80); 0 to 0.75 (*n* = 82); 0.75 to 1.50 (*n* = 47); and >1.50 (*n* = 41). H_FFM_-SDS was likewise divided into five categories: < −1.0 (*n* = 42); −1.0 to −0.5 (*n* = 55); −0.5 to 0 (*n* = 60); 0 to 1 (*n* = 90); and >1.0 (*n* = 44). These cut-offs were selected to produce groups with minimum sample size >40, whilst also distributed as evenly as possible across the range of variability of the trait.

Confidence ellipses were drawn using Piccoli’s software [32], and groups were compared using the Excel function for Hotelling’s t-test.

To control for age, all BIVA outcomes were converted to SDS using Cole’s LMS method (LMS Chart Maker, Medical Research Council, UK), with the two sexes treated separately [33]. This method was previously used to generate SDS for FFM, FM and hydration [22]. The approach provides three outputs: (a) a smoothed median (M or mu) curve which represents how the outcome varies in relation to age; (b) the coefficient of variation (S or sigma), which models the scatter of values around the mean and adjusts for any non-uniform dispersion; and (c) the skewness (L or lambda) which is addressed using age-specific Box-Cox transformation to achieve a normal distribution. Goodness-of-fit was assessed with the Bayesian Information Criterion, adding an extra unit of complexity to the model only if it reduced the deviance by more than Ln(N) units, where N is the sample size.

All SDS outputs were tested for normality, and were normally distributed. Correlation and multiple regression analysis were used to explore associations of BIVA-SDS with body composition SDS, and also BMI-SDS for comparison. BIVA-SDS were compared across categories of FFM-SDS and H_FFM_-SDS. Tolerance elipses were obtained for FFM groups and hydration groups using the software of Piccoli, and compared using Hotelling’s t-test.

## Results

A total of 291 individuals provided data for analysis, 135 boys and 156 girls. Average age was 12.0 (SD 3.7) years, range 4.2 to 19.9 years. There were no significant differences in age or sex ratio across the FFM or H_FFM_ groups.

**Fig. 1:**
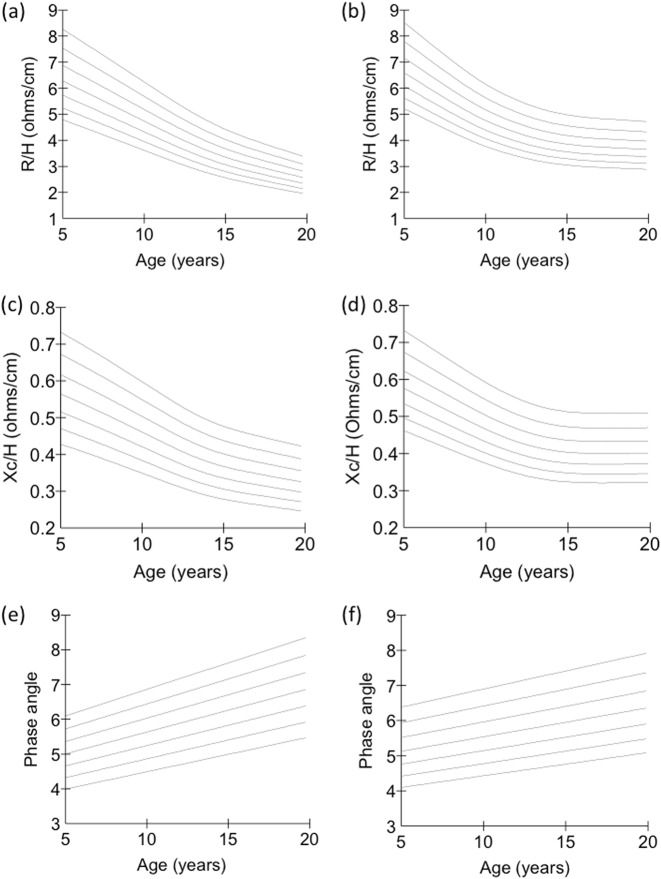
Centile charts for BIVA outputs. Left hand column males, right hand column females

Figure 1 illustrates BIVA ‘growth charts’ for each sex. R/H and Xc/H declined with age in both sexes in curvilinear manner, but the shape of the centiles differed by sex. PA increased in linear manner with age in each sex. The reference data for calculating BIVA SDS are available to download (Supplementary online Datafile 1).

Table 1 shows correlations between BIVA outcomes and body composition measurements, all adjusted for age and sex. PA-SDS was inversely associated R/H-SDS, positively associated with Xc/H-SDS and FFM-SDS, and not associated with FM-SDS or H_FFM_-SDS. R/H-SDS was positively correlated with Xc/H-SDS, and inversely correlated with FFM-SDS, FM-SDS and H_FFM_-SDS. Xc/H-SDS was inversely associated with FFM-SDS, FM-SDS and H_FFM_-SDS. The magnitude of the correlation of BMI-SDS with BIVA outcomes resembled that for FFM-SDS rather than FM-SDS for PA-SDS, but resembled that for FM-SDS rather than FFM-SDS for Xc/H-SDS, and was intermediate between the FFM-SDS and FM-SDS correlations for R/H-SDS. The correlations were very similar when stratified by narrow age ranges (Supplementary online Table 1).

**Table 1:** Correlations between body composition and BIVA SDS

	RH-SDS	XcH-SDS	BMI-SDS	FFM-SDS	FM-SDS	Hydration-SDS
PA-SDS	**−0.32**	**0.40**	**0.20**	**0.29**	0.01	−0.07
R/H-SDS		**0.73**	**−0.72**	**−0.89**	**−0.58**	**−0.32**
Xc/H-SDS			**−0.55**	**−0.65**	**−0.54**	**−0.35**
BMI-SDS				**0.74**	**0.92**	**0.45**
FFM-SDS					**−0.61**	**0.31**
FM-SDS						**0.52**

All correlations in bold significant p<0.0001

PA – phase angle, R/H – height-adjusted resistance, Xc/H – height-adjusted reactance

BMI – body mass index, FFM – Fat-free mass, FM – Fat mass, H_FFM_ – Hydration, SDS – standard deviation score

**Fig. 2:**
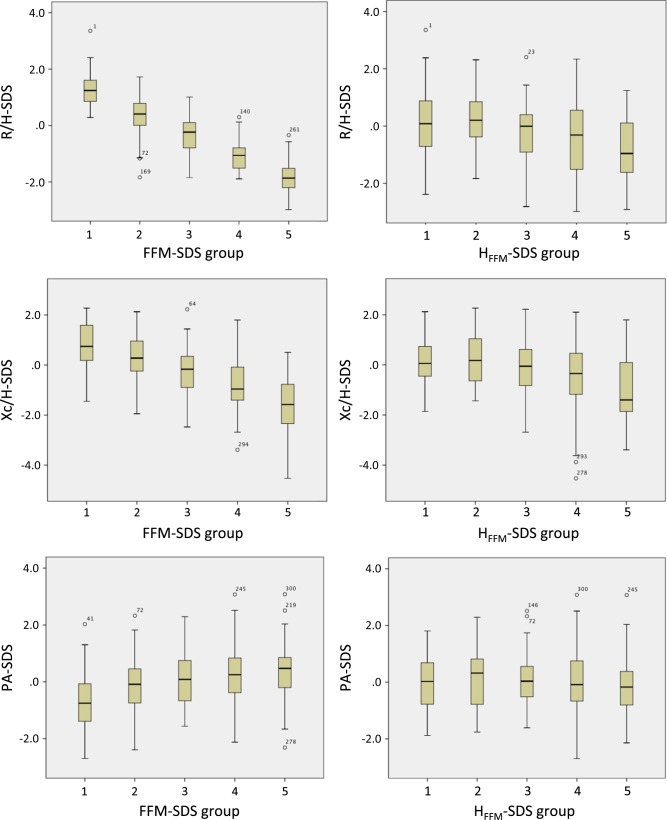
Associations of BIVA-SDS with categories of fat-free mass and hydration. Left hand column males, right hand column females. (FFM – fat-free mass; H_FFM_ – hydration; SDS – standard deviation score). Group contrasts tested by ANOVA with Bonferroni correction (see main text)

Figure 2 presents plots of the three BIVA SDS against groups of FFM-SDS and H_FFM_-SDS. R/H-SDS declined strongly with increasing FFM, with every group-contrast significant (p<0.05) by ANOVA with Bonferroni correction. Likewise, Xc/H-SDS declined with increasing FFM, with all contrasts significant except that between the two lowest FFM groups. For PA-SDS, the low-normal group was significantly different from all other groups, but no other contrasts were significant. R/H-SDS was similar across the first part of the hydration range and then fell, with the highest H_FFM_ group having values significantly different to all other groups, and the fourth-highest group having values different from the two lowest groups. A similar pattern was evident for Xc/H-SDS. PA-SDS showed no significant difference between any of the H_FFM_ groups.

**Fig. 3:**
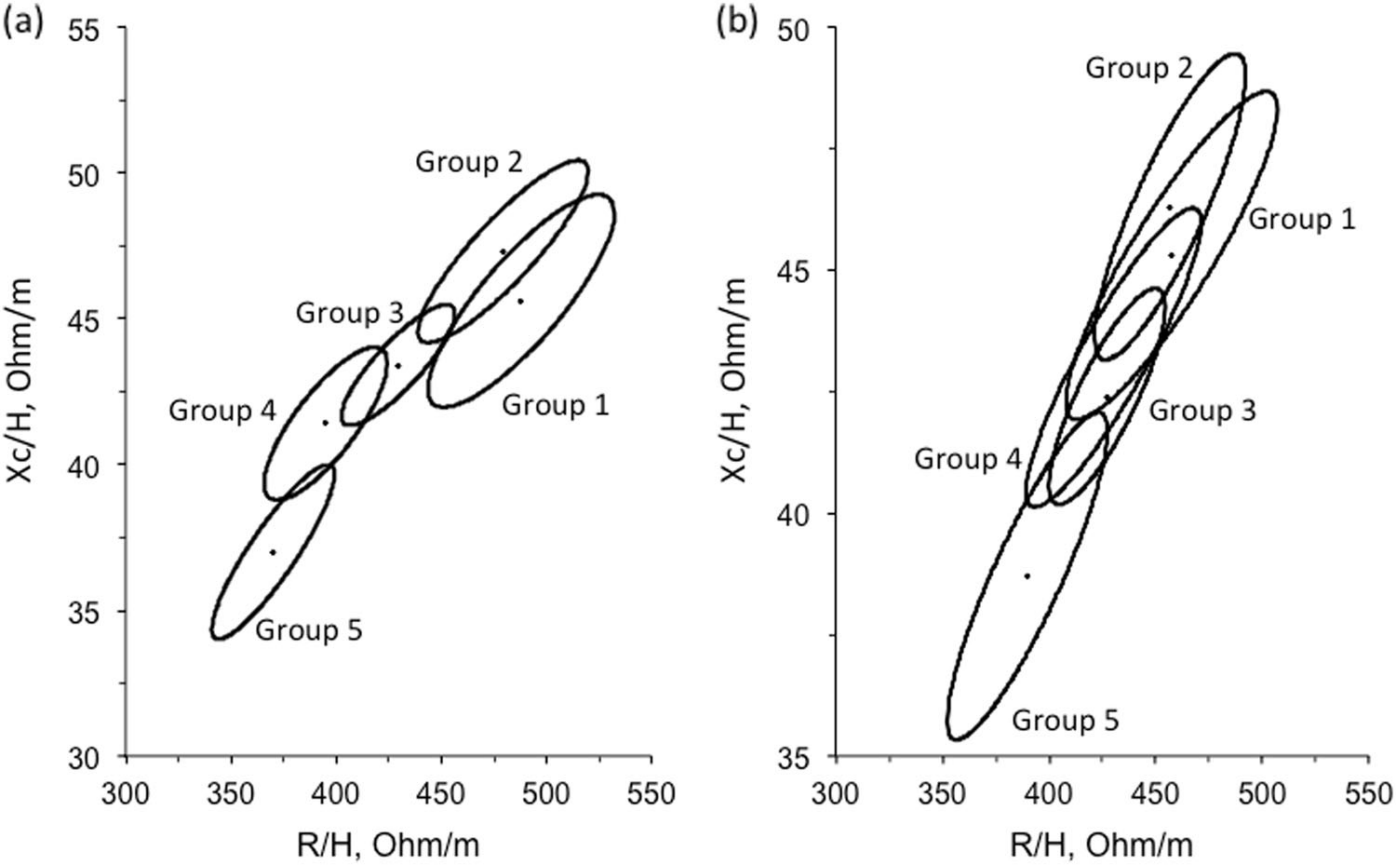
Confidence ellipses by category of **a** fat-free mass and **b** Hydration. (FFM – fat-free mass; H_FFM_ – hydration). Group contrasts tested by Hotelling’s t-test (see main text)

Figure 3 illustrates means and their 95% confidence ellipses for the 5 FFM-SDS and 5 H_FFM_-SDS groups. For FFM groups, differences were found by Hotelling’s t-test between Group 1 and Groups 3–5, while groups 2 and 3 each differed from both group 4 and 5. For hydration groups, differences were found between Group 5 and Groups 1 to 3, and between Groups 2 and 4.

For FFM, the five ellipses were broadly distributed within a single plane, with the exception of the lowest FFM group which appeared displaced to the right. Using linear regression, R/H was a highly significant predictor of Xc/H, explaining 75.5% of the variance. However, when dummy variables for different FFM-SDS groups were entered, none was significant, nor was there any significant interaction between FFM-SDS group and R/H. Thus, there was no evidence that the overall relationship between Xc/H and R/H varied by level of FFM. For hydration, the 5 ellipses lay in the same plane on the graph. When dummy variables for different H_FFM_-SDS groups were entered into the regression model, only Group 5 was significant, and it also showed an interaction with R/H in predicting Xc/H. Thus, individuals with high hydration had significantly lower Xc/H than the other groups for their R/H values.

Table 2 provides multiple regression models for the prediction of FFM-SDS and H_FFM_-SDS from BIVA properties (Table 2). When all three BIVA terms were included, R/H-SDS was the only significant predictor of FFM-SDS, and the model explained 78.8% of the variance. In contrast, both R/H-SDS and PA-SDS were significant predictors of H_FFM_-SDS, but explained only 13.5% of the variance.

**Table 2:** Multiple regression models for the prediction of FFM-SDS and HFFM –SDS from BIVA SDS

Outcome	Predictors	B-coefficient	Standard error	p-value	r^2^
FFM-SDS	Constant	0.013	0.032	0.6	0.787
	R/H-SDS	−0.775	0.192	<0.0001	
	Xc/H-SDS	−0.132	0.184	0.4	
	PA-SDS	0.117	0.163	0.4	
H_FFM-_SDS	Constant	−0.086	0.056	0.12	0.135
	R/H-SDS	−0.840	0.332	0.012	
	Xc/H-SDS	0.488	0.318	0.12	
	PA-SDS	−0.619	0.281	0.028	

PA – phase angle, R/H – height-adjusted resistance, Xc/H – height-adjusted reactance

FFM – Fat-free mass, H_FFM_ – Hydration, SDS – standard deviation score

Finally, explorative regression analysis was conducted under the reverse logic, to test the extent to which variability in body composition parameters (FFM-SDS, FM-SDS and H_FFM_-SDS) could explain variability in BIVA parameters (Table 3). R/H-SDS was negatively associated with FFM-SDS, while there were no independent associations with FM-SDS or H_FFM_-SDS. This model was therefore identical to the equivalent model in Table 2. In contrast, Xc/H-SDS was inversely associated with each FFM-SDS, FM-SDS and H_FFM_-SDS, while PA-SDS was positively associated with FFM-SDS, negatively associated with FM-SDS, but not associated with H_FFM_-SDS. The results for Xc/H-SDS and R/H-SDS indicate that body fat content also predicts variability in some BIVA outcomes.

**Table 3:** Multiple regression models for the prediction of BIVA SDS from body composition SDS

Outcome	Predictors	B-coefficient	Standard error	p-value	r^2^
RH SDS	Constant	−0.035	0.033	0.29	0.790
	FFM-SDS	−0.829	0.033	<0.0001	
	FM-SDS	−0.038	0.033	0.26	
	Hydration-SDS	−0.041	0.036	0.25	
XcH SDS	Constant	−0.051	0.057	0.37	0.454
	FFM-SDS	−0.529	0.057	<0.0001	
	FM-SDS	−0.175	0.058	0.003	
	Hydration-SDS	−0.118	0.062	0.059	
PA SDS	Constant	−0.027	0.059	0.65	0.127
	FFM-SDS	0.388	0.059	<0.0001	
	FM-SDS	−0.165	0.060	0.006	
	Hydration-SDS	−0.103	0.064	0.11	

PA – phase angle, R/H – height-adjusted resistance, Xc/H – height-adjusted reactance

FFM – Fat-free mass, FM – Fat mass, SDS – standard deviation score

## Discussion

Our analysis offers the first opportunity to test whether the assumptions of the Xc/R plots, already supported in studies of extreme changes in body composition and fluid dynamics [12], are further consistent with broader variability in body composition and hydration evident in the general population. We studied children embracing a wide range of age and nutritional status, in order to establish the associations of these parameters with BIVA outcomes, and calculated SDS for each sex separately. We used FFM as an index of cell mass, and measured hydration directly.

We detected correlations of BIVA outcomes with both body composition and hydration, but not in the way predicted by classic BIVA theory [12]. Whereas the theoretical model proposes that BIVA plots are characterised by orthogonal axes indexing variability in hydration and cell mass [5], we found that both sources of variability plotted in the same plane. Increasing R/H and Xc/H were both associated with lower levels of hydration and lower levels of FFM. The theoretical BIVA model therefore behaved as expected for hydration, but not for FFM. However, BIVA parameters explained substantially more variability in FFM than in hydration, possibly because while those overweight tend to have elevated hydration, we may have lacked representation of the lower end of the hydration range.

An exploratory finding was that variability in fatness explained some of the variability in BIVA parameters. Although there is an inherent correlation between the level of FFM and the level of adiposity within children [27], multiple regression analyses indicated an independent contribution of fatness to the variability of BIVA parameters. Whether the magnitude of the correlation of BMI-SDS with BIVA SDS resembled that for FFM-SDS, or that for FM-SDS, varied by BIVA outcome, suggesting that direct measurements of body composition are needed to improve understanding of BIVA variability. These findings may stimulate further development of BIVA theory for paediatric application.

Our results contrast with previous work on individuals with larger degrees of body composition variability or severe fluid perturbations, in which changes in BIVA parameters are associated with variability in both hydration or cell mass [12, 18]. This suggest that there are different ways in which body composition properties relate to BIVA parameters, and that the normal range of variability does not show the same associations as more marked perturbations. It was previously suggested that variability in BIVA parameters in children might be better explained by taking into account body shape variability [34]. This was recently supported for adults [35], improving the prediction of tissue masses, hence this represents an important avenue for future research on BIVA in children and might resolve the poor fit between model and data we describe here.

In adults, substantial variability in PA was explained by FFM [11]. However, removing the contributions of age and sex, we found that only a small amount of variability in PA was explained by FFM in children and adolescents, with fat mass also contributing, but hydration not significant. Our definition of FFM incorporates any water content of adipose tissue, hence associations of BIVA parameters with fat mass are not due to variability in adipose tissue hydration, though BIA models can address this [36]. PA has proven very valuable in predicting clinical prognosis across diverse diseases [10], but it is complex to interpret as it has been indirectly associated with both tissue magnitudes and properties such as cell membrane status, in contrast to R and Xc which relate directly to water compartments, and the capacitive opposition of cell membranes to current flow, respectively. Further work is therefore necessary to improve understanding of exactly what PA indexes at the physiological level in younger age groups, and might also consider whether the optimal frequency for collecting data differs from 50 kHz in children.

The strengths of our analysis included a large sample size, in which we were able to adjust BIVA parameters for age and sex by making SDS. We were able to pursue the same approach for our body composition predictors, allowing us to conduct all analyses independent of age and sex. This is important, because numerous previous studies have demonstrated that BIVA parameters vary with both age and sex.

Another strength was the availability of objective data on both hydration and FFM, a useful proxy for cell mass. Although both outcomes derived from the same 4-component model, and hence might be affected by common measurement error, we have previously demonstrated that FFM-SDS by the 4-component model correlates very strongly with independent measurements by DXA. An independent evaluation of hydration is more difficult, hence the correlation with BIVA parameters in this study provides new supporting evidence although only a small proportion of the hydration variance was explained by them.

A limitation of our analysis is that we did not have direct data on cell mass, nevertheless FFM should act as a valid proxy. Another limitation is that we restricted our analysis to individuals of European ancestry, hence our findings might not apply to other populations. However, although both body composition [19–21] and BIVA ellipses vary with ethnicity [14], we are unaware of any reason why associations between these traits should vary substantially by ethnicity. Finally, although the sample size was relatively high, we had relatively few individuals characterised by very low FFM, hence such individuals merit further investigation.

In summary, our study failed to link BIVA parameters with body composition outcomes as expected on the basis of the theoretical model of Piccoli. Normal variability in hydration and FFM does not fit the model in the same way as larger levels of variability, associated with extremes of body composition or illness. Our novel approach, generating SDS for BIVA outcomes, may help apply the BIVA approach in children and adolescents in future, as it removes the need for age-specific reference data and provides a valuable approach for ranking individuals.

## Electronic supplementary material

Supplementary online Table 1

BIVA reference 2018
