# Supplementary material for: Bio-electrical impedance vector analysis: testing Piccoli’s model against objective body composition data in children and adolescents
Source: Eur J Clin Nutr. 2018 Aug 30;73(6):887–95. doi: 10.1038/s41430-018-0292-x (PMC6760620; doi:10.1038/s41430-018-0292-x)
Supplement: Supplementary file 1 — Supplementary online Table 1 [file 41430_2018_292_MOESM1_ESM.docx]

**Supplementary online Table 1. Correlations between BIV-SDS and body composition SDS stratified by age group**

| Age group | R/H | | Xc/H | | PA | |
| --- | --- | --- | --- | --- | --- | --- |
|  | FFM | H_FFM_ | FFM | H_FFM_ | FFM | H_FFM_ |
| Whole sample (n=291) | **-0.89** | **-0.32** | **-0.65** | **-0.35** | **0.29** | -0.07 |
| 4-7.9 y (n=49) | **-0.89** | -0.04 | **-0.55** | 0.01 | **0.33** | 0.04 |
| 8-10.9 y (n=64) | **-0.89** | **-0.32** | **-0.71** | **-0.34** | 0.13 | -0.09 |
| 11-12.9 y (n=58) | **-0.91** | **-0.32** | **-0.73** | **-0.40** | **0.32** | -0.13 |
| 13-15.9 y (n=78) | **-0.90** | **-0.36** | **-0.57** | **-0.40** | **0.46** | -0.04 |
| 16-19.9 y (n=42) | **-0.88** | **-0.36** | **-0.74** | **-0.36** | 0.08 | -0.09 |

Values are correlation coefficients (those in bold are significant p<0.05)

FFM – fat-free mass; H_FFM_ – hydration of FFM

All correlations tested on BIVA-SDS, FFM-SDS and H_FFM_-SDS
